# Complete sequences of conjugal helper plasmids pRK2013 and pEVS104

**DOI:** 10.17912/micropub.biology.000882

**Published:** 2023-07-13

**Authors:** Ashley Hall, Timothy Donohue, Jason Peters

**Affiliations:** 1 Great Lakes Bioenergy Research Center, University of Wisconsin-Madison, Madison, Wisconsin, United States; 2 Wisconsin Energy Institute, University of Wisconsin-Madison, Madison, Wisconsin, United States; 3 Department of Bacteriology, University of Wisconsin–Madison, Madison, Wisconsin, United States; 4 Pharmaceutical Sciences Division, School of Pharmacy, University of Wisconsin–Madison, Madison, Wisconsin, United States; 5 Department of Medical Microbiology and Immunology, University of Wisconsin–Madison, Madison, Wisconsin, United States; 6 Center for Genomic Science Integration, University of Wisconsin–Madison, Madison, Wisconsin, United States

## Abstract

We present the complete sequences of two commonly used conjugal helper plasmids: pRK2013 and pEVS104. These sequences will enable engineering of custom helper plasmids, for example, with different antibiotic markers or origins of replication. We provide both sequence information and plasmid maps to aid future engineering efforts.

**Figure 1.**
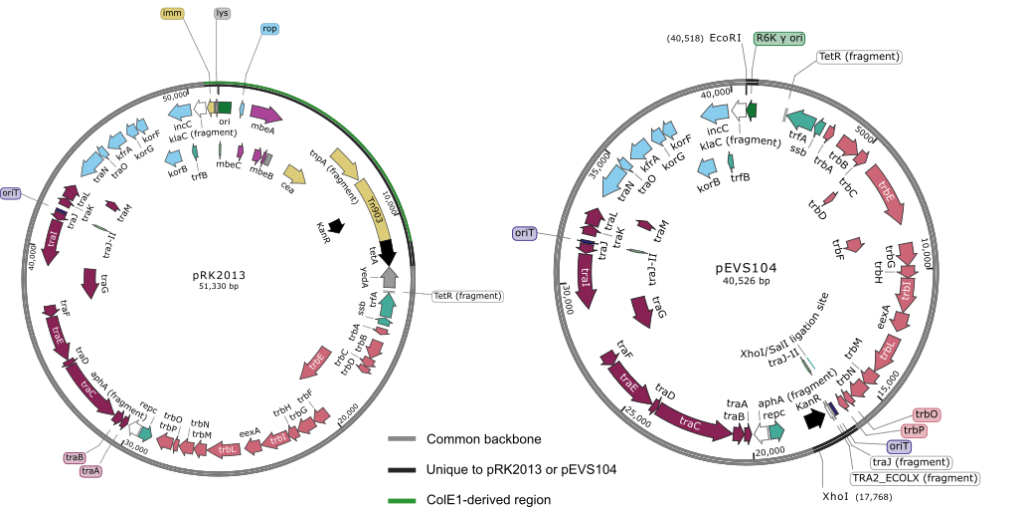
Plasmid maps for each pRK2013 and pEVS104.

## Description

Genetic manipulation of diverse bacteria requires facile transfer of engineered DNA, often in the form of plasmids. Conjugation is a common approach to transfer plasmids from *Escherichia coli* to bacteria of interest. Genes required for conjugation are often taken from a broad host range conjugal plasmid, such as pRK2, and either inserted into the chromosome or are present on a replicating “helper” plasmid (Motallebi-Veshareh *et al.* 1990; Zatyka and Thomas 1998). The IncP-type helper plasmid pRK2013 and its derivatives are capable of mobilizing plasmids into diverse recipient bacteria including *Acinetobacter* (Ely 1985; Towner 1991) *, Agrobacterium* (Shaw 1995) , *Bacillus* (Heinze *et al.* 2018), *Burkholderia* (Malott *et al.* 2012), *Campylobacter* (Akiba *et al.* 2006), *Caulobacter* (Ely 1985) *Chroococcidiopsis,* (Billi *et al.* 2001), *Desulfovibrio* (Argyle *et al.* 1992), *Halomonas* (Ventosa *et al.* 1998), *Nostoc* (Cohen *et al.* 1994) *, Pseudomonas* (Zylstra *et al.* 1988), *Rhizobium* (Ditta *et al.* 1980; Bullerjahn and Benzinger 1984; Ely 1985), *Rhodobacter* (Ma *et al.* 2018), *Rhodospirillum* (Saegesser *et al.* 1992), *Salmonella* (Rainey *et al.* 1997) *Sphingomonas* (Kobayashi *et al.* 2016), *Streptomyces* (Thomas *et al.* 2003), *Vibrio* (Datta *et al.* 1984) *, Xanthamonus* (Turner *et al.* 2009) *Yersinia* (Biedzka-Sarek *et al.* 2005), and *Zymomonas* (Conway *et al.* 1987), among others. Although use of pRK2013 is widespread (>2,000 citations as of 2/2023), the complete plasmid sequence is not available. Here, we provide the sequence of pRK2013, an RK2-derived plasmid with a ColE1 origin of replication, and pEVS104, an improved derivative of pRK2013 with an R6Kγ origin of replication.

Sequence and assembly of plasmids pRK2013 and pEVS104 agree with previous descriptions of plasmid construction (Figurski and Helinski 1979; Ditta *et al.* 1980; Stabb and Ruby 2002). Most of the plasmid backbone is shared between pRK2013 and pEVS104 (Fig 1AB, gray backbone) and is derived from the IncPα plasmid pRK2. This backbone contains genes for conjugal transfer and mating pair formation (Tra1 and Tra2 regions; *tra* and *trb* genes) (Pansegrau *et al.* 1994; Zatyka and Thomas 1998). Transcriptional regulators, plasmid partitioning, and plasmid maintenance genes ( *korAB* , *incC* , *kfrABC* ) are also present. The *kilA* locus ( *klaABC* genes) of plasmid RK2 was largely removed, however a fragment of the *klaC* gene remains (Goncharoff *et al.* 1991).

The pEVS104 variant of pRK2013 has improved properties as a helper plasmid (Stabb and Ruby 2002) . pEVS104 lacks a transposon found in pRK2013 that can mutagenize the recipient strain. Furthermore, pEVS104 cannot replicate in recipient bacteria; pRK2013 replicates via a ColE1 origin, but pEVS104 uses an R6Kγ origin that can only replicate in engineered *E. coli* donor cells containing a *pir* gene. The ColE1-derived plasmid segment (black and green backbone, Fig 1A) containing the ColE1 origin of replication, Tn903, and the genes encoding colicin E1 ( *cea* ) and its cognate immunity gene ( *imm* ) were removed in constructing pEVS104 (Stabb and Ruby 2002) . The proximal *tetA* gene was also removed (Fig 1A black backbone). pEVS104 has two insertions relative to pRK2013: an R6K γ origin of replication and a second *oriT* with a linked *kanR* gene, used to select for maintenance of pEVS104 ( Fig. 1B, black backbone ).

**Nucleotide sequence accession number**

pRK2013 has GenBank accession number OQ536579 . pEVS104 has GenBank accession number OQ536580 . Raw reads are deposited on SRA under BioProject PRJNA955763 .

## Methods

Plasmid DNA was extracted from *E. coli* strains sJMP4003 and sJMP4007 using the PureLink HiPure Plasmid Filter Midiprep Kit (K210014, Invitrogen) with 100mL of saturated culture. Sequencing and assembly of plasmid sequences were performed by Plasmidsaurus (Eugene, OR, USA) with an Oxford NanoporePromethION on an R10.4.1 flowcell with primer-free Kit 14 chemistry. Raw fastq reads, assemblies, and annotations performed with pLannotate (McGuffie and Barrick 2021) were received from Plasmidsaurus (Eugene, OR, USA).To uncover genes that were missing in our automated annotation, we identified ORFs using SnapGene Pro v6.2.1 (www.snapgene.com) and used BLASTp to assign a putative gene function (Default BLASTp parameters searching Standard database) (Altschul *et al.* 1990). pRK2013 coverage was ~30X (123 reads), pEVS104 coverage was ~ 440X (527 reads).
